# Beyond Weight Loss: Holistic Impacts of a Digital Weight Management Programme Integrating Tirzepatide

**DOI:** 10.7759/cureus.102817

**Published:** 2026-02-02

**Authors:** Lena M Sikorska, Vivian N Liu, Hans Johnson, Earim Chaudry, Daniel Reisel, Ashley K Clift, David R Huang

**Affiliations:** 1 Clinical Innovation and Research, Menwell Limited (Voy), London, GBR; 2 Digital Health and Care, University of Bristol, Bristol, GBR; 3 Obstetrics and Gynaecology, Elizabeth Garrett Anderson (EGA) Institute for Women's Health, University College London, London, GBR

**Keywords:** binge eating disorder, cardiometabolic markers, digital health intervention, digital weight management, glp-1 receptor agonist, mental health outcomes, obesity treatment, tirzepatide

## Abstract

Introduction

Recent pharmacological advancements, such as tirzepatide, a dual GIP/GLP-1 receptor agonist, offer new therapeutic options, delivering substantial weight loss with potential mental health and well-being benefits. Digital weight loss services (DWLS) that combine anti-obesity medication with nutritional and behavioural support are increasingly used, but real-world evidence remains limited as clinical trial outcomes may not reflect routine practice. This service evaluation assessed 12-month outcomes of individuals enrolled in Voyager, a pilot programme delivered by the Voy DWLS.

Methods

UK residents (n=60) aged 18-65 with body mass index (BMI) ≥30 kg/m², naive to prior anti-obesity medication. Participants attended the Voyager programme between July 2024 and August 2025, comprising tirzepatide treatment and digital interventions (health coaching, side effects tracking, clinical support, app access, and webinars). The primary outcome was the percentage total body weight loss (WL) from baseline to 12 months. Secondary outcomes included anthropometric measures, patient-reported outcomes (Binge Eating Scale (BES) and Patient Health Questionnaire-9 (PHQ-9) scores), blood pressure, side effects, and laboratory results. Statistical analyses employed descriptive statistics, paired t-tests, and linear mixed-effects models.

Results

Participants were predominantly female (90%, n=54), with a mean age of 44.6±11.1 years and baseline BMI of 37.1±6.2 kg/m². At 12 months, mean WL reached 22.7% (95% CI 20.92-24.48; p<0.001). Waist and hip circumference, and waist-to-hip ratio, declined significantly. BES scores fell by 18 points (95% CI 14.5-21.67) and PHQ-9 by 3.06 points (95% CI 2.26-3.87). Mean systolic and diastolic blood pressure decreased by 9.07 mmHg (95% CI 5.52-12.61) and 4.90 mmHg (95% CI 2.53-7.26), respectively (all p<0.001). Nausea was the most common side effect and decreased over time. Significant longitudinal improvements occurred across cardiometabolic markers, including glycated haemoglobin (HbA1c), low-density lipoprotein (LDL) cholesterol, total cholesterol, and cholesterol/high-density lipoprotein (HDL) ratio; all five participants with prediabetes normalised HbA1c by 12 months.

Conclusion

The Voyager programme integrating tirzepatide achieved clinically meaningful weight loss and broad metabolic and psychological improvements over 12 months, supporting integrated pharmacological-digital approaches to obesity care.

## Introduction

Background

Obesity is a chronic, multifactorial disease affecting more than one in four adults and is associated with increased risks of type 2 diabetes, coronary heart disease, stroke, and certain cancers [1, 2]. It also contributes to reduced quality of life (QoL) and a growing pressure on the healthcare system, with annual costs estimated from economic productivity losses of £8.9 billion in the United Kingdom (UK) per year [3].

In the context of obesity’s growing healthcare and economic burdens, recent developments in pharmacological treatments, such as tirzepatide, a dual glucose-dependent insulinotropic polypeptide (GIP) and glucagon-like peptide-1 (GLP-1) receptor agonist (GIP/GLP-1 RA), represent novel opportunities for weight management [4]. Clinical trials have demonstrated that tirzepatide and other anti-obesity medications achieve greater weight loss than non-pharmacological approaches; as is illustrated in the SURMOUNT-CN study, 10mg tirzepatide resulted in 11.3% (95% CI 8.3% to 14.3%, p<0.001) more weight loss than placebo over 53 weeks [5].

Emerging research also indicates that anti-obesity medications like tirzepatide may not only aid weight reduction but also offer positive mental health and well-being effects. In their systematic review of studies of varying follow-up duration, Radkhah and colleagues found that patients receiving anti-obesity medication experienced significantly greater improvements in their binge eating symptoms compared with controls, with a mean reduction of 8.14 points on the Binge Eating Scale (BES) (95% CI 3.15 to 13.13, p<0.01). They also reported significantly greater weight loss (3.81 kg) (95% CI 2.49 to 5.14, p<0.01) and reductions in body mass index (BMI) (1.48 kg/m²) (95% CI 0.90 to 2.06, p<0.01) and waist circumference (3.14 cm) (95% CI  1.11 to 5.16, p<0.01), suggesting that anti-obesity medications play a promising role in the management of binge eating disorder [6].

Evidence also indicates that anti-obesity medications may confer improvements in depressive symptoms. In another systematic review, Chen and colleagues found that patients receiving anti-obesity medications experienced significantly greater improvements in depressive symptoms compared to those in the control group, although the included studies used different depression rating scales, with a standardised mean difference of -0.12 (95% CI -0.21 to -0.03, p<0.01) [7]. However, as the current research is constrained by limited follow-up and a lack of studies focused specifically on tirzepatide, further real-word research is necessary to explore the potential effects of GIP/GLP-1 and GLP-1 medications on binge eating-type behaviours [6, 8-10] or mental health symptoms [7], which can have a further detrimental impact on physical well being, and ultimately, attenuating QoL.

There is an emerging consensus within the field that GIP/GLP-1 RA should be used together with comprehensive care, including behavioural and dietary support, to maximise the benefits and minimise the risks of treatment [11]. Consequently, digital weight management programmes offering anti-obesity medication within wider packages of non-pharmacological (dietary and behavioural) support are becoming increasingly popular. Such integrated digital weight loss service (DWLS) models may improve metabolic health, adherence to medication and overall weight loss results [12]. In their recent retrospective cohort analysis, Johnson and colleagues showed that, at 11 months, digital engagement with a weight management programme was associated with 26.5% relative increase in weight loss (21.5% engaged vs. 17.0% non engaged, p<0.001), faster milestone achievement, and comparable safety profiles [13].

Objectives

This retrospective cohort service evaluation aims to assess the real-world effectiveness of a 12-month weight management programme, a DWLS, combining pharmacological treatment with tirzepatide and digital health tools, with a focus on overall holistic potential benefits. The programme is designed to help participants achieve clinically meaningful weight loss, improved health, and enhanced quality of life through the combined use of a comprehensive digital intervention with behavioural and lifestyle support and a pharmacological adjunct. Given that existing real-world evidence on such interventions often focuses solely on weight outcomes, insight into their broader psychological and well-being effects remains limited. 

This study seeks to address this gap by evaluating both primary outcomes of 12-month weight loss and secondary outcomes capturing holistic benefits.

These secondary outcomes include changes in patient-reported wellbeing, quality of life, and other clinical and anthropometric outcomes, particularly GIP/GLP-1 RAs potential role in alleviating binge eating and depressive symptoms.

## Materials and methods

Study design and setting

This study retrospectively evaluated the Voyager programme within the Voy digital weight loss service in the United Kingdom (UK). This programme comprised two components: a pharmacological treatment with tirzepatide and complementary digital interventions, delivered over a 12-month period. The programme was delivered fully remotely (asynchronously via the Voy app and through teleconsultations with health and behavioural coaches, as well as the clinical team) to patients in the UK between July 2024 and August 2025.

The digital component of the programme delivered individualised treatment plans with a focus on education and behaviour change, supported by virtual care and enabled by a comprehensive, end-to-end healthcare operating system. In this specific cohort, individuals were provided with equipment such as Bluetooth weight scales, a blood pressure monitor, and prospectively contributed data via questionnaires (detailed below).

Individualised Treatment Plans

Each participant received a specialist-led care plan, tailored to their evolving health needs. Participants were encouraged to share their experience with the clinical team, as well as track their side effects (weekly), health metrics such as weight (bi-weekly), and patient-reported outcomes (monthly) through online questionnaires and the Voy app.

Side effect responses were reviewed by a clinician of the Voyager DWLS and used to direct clinical management plans. Support was offered either in the form of lifestyle advice, medication adjustments or supplementary medications when required, ensuring safe, responsive, and effective plans.

Education and Behaviour Change

Recognising the benefit of combining pharmacological treatment with digital evidence-based support [12], the programme delivered a blended digital-plus-human programme of education and coaching.

Participants received personalised health coaching with professionals trained based on the principles of Social Cognitive Theory, Trans-Theoretical Model, Theory of Planned Behaviour, and Self-determination Theory [14, 15]. Sessions were delivered flexibly through video or phone calls and supplemented with ad-hoc text messages. Coaches provided feedback, motivation, and nutritional guidance, with a focus on goal setting and micro-habits to promote lasting behavioural change. The health coaching support involved fortnightly video calls, unlimited message check-ins, with all sessions being recorded. In addition, to foster community engagement, peer learning and ongoing motivation, participants were invited to attend interactive live webinars. The sessions covered topics such as programme orientation, tirzepatide injection training, movement guidance, and side effects management.

Virtual Care

The clinical team offered participants support online, via email and the Voy in-app chat. They provided responses to clinical safety issues related to medication administration or side effects. Clinical staff promptly addressed patient concerns and were available for on-demand teleconsultations. This omni-channel care model offered patients immediate reassurance, rapid intervention, and consistent continuity of care.

The cohort’s digital component was accessible via smartphone or laptop. The Voy app enabled patients to track health metrics, log meals, access dynamic educational weight management content, request flexible medication titration, and contact both clinical and customer support teams.

Participants

Participants were recruited through social media channels (primarily Meta), invited to join the Voyager programme and subsequently registered via the Voy website. During the registration process, participants consented to Voy’s terms and conditions, which included a general provision allowing the use of anonymised customer information for research and evaluation purposes.

To be eligible for this service evaluation, participants were required to be UK residents aged between 18 and 65 years old with a body mass index (BMI) above or equal to 30 kg/m², and no prior tirzepatide use. Additional inclusion criteria included digital literacy and access to a smartphone or laptop, given the digital format of the programme. Exclusion criteria comprised pre‑existing type 1 or type 2 diabetes mellitus, active malignancy, severe renal or hepatic impairment, pregnancy, and previous bariatric surgery or endoscopic intervention for obesity. Baseline assessments were completed for all participants. Follow‑up completion varies by analyte.

Variables

Primary Outcome

The primary outcome was the percentage total body weight loss from baseline to 12 months, recorded via self-reported questionnaires. Body weight was measured to the nearest 0.1 kg using calibrated digital scales with participants in light clothing and without shoes, and validated by a health care professional (HCP) remotely. BMI was calculated as weight in kilograms (kg). divided by height in metres squared (kg/m²).

Secondary Outcomes

Through the self-reported questionnaires, patients also submitted other anthropometric measures (waist circumference and hip circumference), information about side effects, blood pressure, and patient-reported outcomes (through validated mental health questionnaires, BES, and PHQ-9 scores) [16, 17]. The PHQ-9 is a public domain instrument requiring no permission for use. A questionnaire asked participants to rate how much their weight affects their self-confidence and productivity on a scale of 1 to 5 (1= never, 5 = always).

Demographic information, including age, gender, ethnicity, relationship status, and employment status, was collected at baseline via a structured questionnaire. Medical history was self‑reported at baseline and included hypertension, hypercholesterolaemia, obstructive sleep apnoea, cardiovascular disease, polycystic ovary syndrome, fatty liver disease, thyroid disease, asthma, osteoarthritis, and chronic back pain. Lifestyle factors, including physical activity frequency, alcohol consumption, and smoking status, were also assessed using standardised questionnaire items.

Participants completed a bespoke questionnaire assessing weight-related behaviours and psychological well-being at baseline and 12 months. The questionnaire was developed internally through collaboration between behavioural specialists and clinicians to evaluate key domains relevant to weight management: eating behaviours (food cravings, sense of self-control), psychological impact (self-confidence, body image satisfaction, health concerns), functional impairment (social interactions, shopping for clothes, energy levels, exercise capacity, physical comfort), and wellbeing (sexual life, ability to relax, concentration). Items used a 5-point Likert scale (1=never, 5=always). While not a validated psychometric instrument, this questionnaire provided clinically relevant insights into behavioural and psychological changes during the programme.

Venous blood samples were collected every three months and processed in a United Kingdom Accreditation Service (UKAS)-accredited laboratory according to standard clinical protocols. Measures were obtained at baseline and at 3, 6, 9, and 12 months post‑enrolment. Assays included HbA1c, total cholesterol, LDL, HDL, total cholesterol-HDL ratio, T4, TSH, and ferritin. Biomarker values were categorised according to UK National Health Service (NHS) reference ranges.

Statistical methods

Data handling and initial processing utilised Microsoft Excel (Microsoft, Redmond, Washington), with statistical analyses in R statistical software (version 4.3.0).

Biomarker trajectories were visualised as individual patient spaghetti plots overlaid with population mean trajectories and 95% confidence bands. Distributional shifts over time were illustrated using ridgeline density plots. Patient movement between clinical categories across five pre-selected time points (baseline, 3, 6, 9, 12 months) was shown using alluvial (Sankey) diagrams.

Descriptive statistics were used to summarise the study sample, including means with standard deviations (SD), or counts and percentages (%). Trends in biomarkers from baseline were analysed using: 1) paired t-tests to identify changes at 3, 6, 9 and 12 months; and 2) linear mixed models, with mean values estimated (with 95% confidence intervals) at monthly intervals or at 3, 6, 9 and 12 months, depending on the cadence of data collection for individual outcomes. 

Analyses requiring paired observations, including paired t‑tests and categorical transitions, were restricted to complete pairs for the relevant time points. In mixed models, missing data were handled implicitly under the ‘missing at random’ (MAR) assumption without imputation of data (e.g. missing blood result at month ‘X’ for a given patient). The threshold for clinical significance was set as a two-sided p<0.05.

For participants classified at baseline as higher risk, analyses examined the following trend metrics: proportion crossing the threshold to a healthy category at 12 months; proportion demonstrating clinically meaningful change; and proportion demonstrating any reduction from baseline. Categorical analyses were restricted to participants with both baseline and 12‑month measurements.

Ethical considerations

This work comprised a retrospective, secondary analysis of routinely collected programme service evaluation data. All participants provided informed consent at enrolment. The study was conducted in accordance with the Declaration of Helsinki and UK research governance frameworks. The study was conducted as per an ethical approval from the University College London REC (ID: 2025-0906-775).

## Results

Participants characteristics

A total of 60 patients were included in this service evaluation. They were predominantly female (90%, n=54), with a mean age(±SD) of 44.6±11.1 years old, and most self-identified as White British/Irish (78.3%, n=47). The cohort had a mean (±SD) baseline BMI of 37.1±6.1 kg/m2. See summary baseline demographics and characteristics, including anthropometric measures, lifestyle factors and blood pressure values (Table 1).

**Table 1:** Baseline characteristics of study participants Voyager programme service evaluation cohort baseline demographic characteristics (N = 60). ¹Data presented as mean ± SD; median (QR) for continuous variables; n (%) for categorical variables. United Kingdom (UK) alcohol guidelines: low risk ≤14 units/week, increasing risk 15-35 units/week, higher risk >35 units/week. ²Ethnicity categories were combined to minimise the risk of statistical disclosure. ³Small cell counts have been suppressed for disclosure control purposes. BMI - body mass index; PHQ-9 - Patient Health Questionnaire-9; BES - Binge Eating Scale; PCOS - polycystic ovary syndrome; TSH - thyroid-stimulating hormone; HbA1c - glycated haemoglobin; LDL - low-density lipoprotein; HDL - high-density lipoprotein; T4 - free thyroxine

Characteristic	Value^1^
Demographics	
Total patients, n	60
Age, years	44.6 ± 11.1; 43.5 (35.0-54.0)
Gender, n (%)	
Female	54 (90.0%)
Male	6 (10.0%)
Ethnicity, n (%)^2^	
White - British/Irish/any other White background	51 (85.0%)
Black or Black British - African/Caribbean	<5^3^
Asian or Asian British - Indian/Pakistani/Any other Asian background	<5^3^
Mixed - White and Black African/Caribbean	<5^3^
Anthropometric measurements	
Height, cm	167.1 ± 9.0; 165.0 (162.6-173.0)
Weight, kg	103.4 ± 17.7; 100.1 (89.8-116.8)
BMI, kg/m2	37.1 ± 6.2; 34.4 (32.6-41.4)
Waist circumference	110.35 ± 15.17; 109 (98-121)
Hip circumference	121.48 ± 14.02; 118.50 (11-127)
Waist-to-hip ratio	0.92 ± 0.14; 0.88 (0.84-0.96)
Patient-reported outcomes	
PHQ-9	5.07 ± 4.26; 4 (1-8.25)
BES	22.3 ± 8.02, 22 (18.0-27.5)
Pre-existing comorbidities, n (%)	
High blood pressure	5 (8.3%)
High cholesterol	<5^3^
Obstructive sleep apnoea	<5^3^
Cardiovascular disease	<5^3^
Asthma	<5^3^
Osteoarthritis	5 (8.3%)
Chronic back pain	5 (8.3%)
PCOS	<5^3^
Fatty liver disease	<5^3^
Lifestyle factors	
Physical activity level	
Daily	5 (8.3%)
Occasionally (1-2 times a week)	24 (40.0%)
Rarely or never	10 (6.7%)
Regularly (3-4 times a week)	21 (35.0%)
Alcohol consumption (UK Guidelines), n (%)	
Increasing risk (15-35 units/week)	<5^3^
Low risk (1-14 units/week)	28 (46.7%)
None	29 (48.3%)
Smoking status, n (%)	
Current smoker	<5^3^
Ex-smoker	12 (20.0%)
Never smoked	33 (55.0%)
Not reported	11 (18.3%)
Relationship status, n (%)	
Divorced	<5^3^
Engaged	<5^3^
In a relationship	13 (21.7%)
Married	28 (46.7%)
Single	16 (26.7%)
Employment status, n (%)	
Employed full-time/part-time	45 (75.0%)
Other	<5^3^
Retired	<5^3^
Self employed	8 (13.3%)
Unemployed	<5^3^
Blood pressure	
Systolic blood pressure	124.69 +/- 10.39; 123 (118-130.25)
Diastolic blood pressure	77.65 +/- 7.72; 77 (72.75-81.5)
Pulse	73.40 +/- 8.83; 72.5 (67.5-78.5)
Laboratory blood results	
TSH, mIU/L	2.13 ± 1.26; 1.88 (1.27-2.50)
HbA1c, mmol/mol	35.6 ± 4.9; 36.1 (32.1-38.8)
Total cholesterol, mmol/L	5.73 ± 1.40; 5.56 (4.96-6.13)
LDL cholesterol, mmol/L	3.57 ± 1.14; 3.47 (2.91-4.08)
HDL cholesterol, mmol/L	1.43 ± 0.43; 1.42 (1.18-1.67)
Total cholesterol/HDL Ratio	4.36 ± 1.71; 3.89 (3.53-4.79)
Free T4, pmol/L	16.1 ± 2.3; 15.7 (14.5-17.5)
Ferritin, μg/L	108.3 ± 102.0; 86.3 (46.1-145.4)

Regarding data collection: baseline data collection for anthropometrics was complete for all individuals. Forty-three out of 60 individuals (71.67%) reached 12 months of follow-up. 54-57 participants contributed baseline samples depending on the biomarker, and 42-46 participants contributed biomarker samples at 12‑month follow-up. Complete paired data (baseline and 12‑month measurements) were available for 42 participants for lipid markers and HbA1c.

Weight loss outcomes

**Figure 1:**
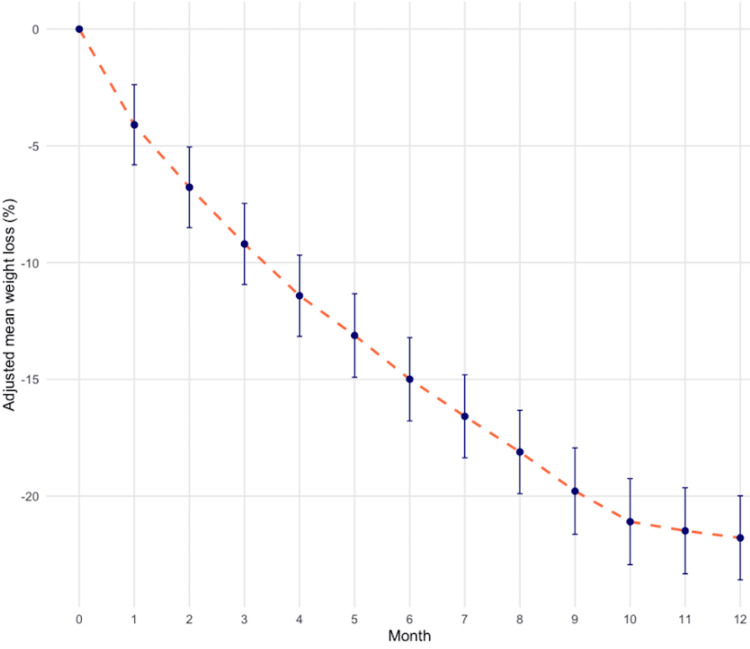
Weight loss outcomes. Adjusted mean percentage weight loss over 12 months among the cohort. Red dashed line: mean trajectory of weight loss; error bars: 95% confidence intervals. Data shows a progressive reduction in body weight, reaching 22.7% (95% CI 20.92% to 24.48%, p<0.001) mean weight loss at month 12.

The mean percentage total body weight loss at 12 months was 22.7% (95% CI 20.92% to 24.48%, p<0.001) (Figure 1).

Other anthropometric outcomes

**Figure 2:**
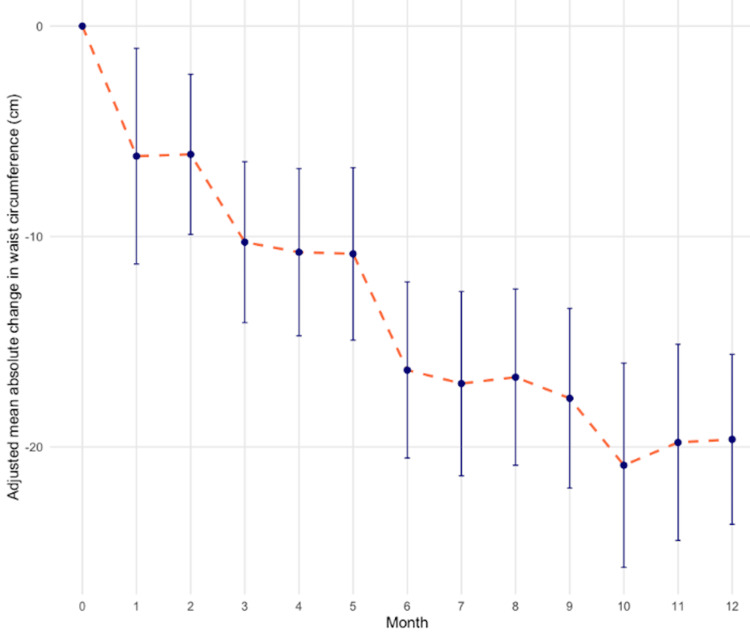
Change in waist circumference. Adjusted mean absolute change in waist circumference (cm) over 12 months among the cohort. Red dashed line: mean trajectory of change; error bars: standard deviations. Data shows a progressive reduction in waist circumference, reaching 21.47cm (95% CI 19.79 to 24.15, p<0.01) mean change at month 12.

Secondary anthropometric outcomes showed a significant decrease in waist and hip circumference, as well as waist:hip ratio (Figure 2). At 12 months, the mean decrease in waist circumference was 21.47cm (95% CI 19.79 to 24.15, p<0.001) and in hip circumference this was 16.17cm (95% CI 13.26 to 19.07, p<0.001). Overall, by 12 months, the mean waist-to-hip ratio was 0.86, representing a mean reduction of 0.06 from baseline (95% CI 0.03 to 0.08, p<0.01). From the mixed models, women had a mean baseline waist-to-hip ratio of 0.90 (95% CI: 0.88 to 0.93) and reached a mean waist-to-hip ratio of 0.85 (95% CI: 0.82 to 0.87) at 12 months, while men had a mean baseline waist-to-hip ratio of 1.08 (95% CI: 1.00 to 1.15) and reached a mean of 0.95 (95% CI: 0.84 to 1.00) at 12 months.

Patient-reported outcomes

The mean baseline BES was 22.3 ± 8.02 points, and the mean baseline PHQ-9 score was 5.07 ± 4.26 points. At baseline, participants’ mean ratings of how often their weight affects their confidence was 4.42/5, and their ratings of how often their weight affects their productivity was 3.2/5.

Patients experienced improvements in their binge eating and depression scores. At 12 months, BES was reduced by 18 points (95% CI 14.5 to 21.67, p<0.001). There was also a significant reduction in PHQ9 scores - an average reduction was 3.06 points (95% CI 2.26 to 3.87, p<0.001). At 12 months, on average, participants’ mean ratings of how often their weight affects their confidence was 1.78/5, and rated how often their weight affects their productivity as 1.43/5 (both significant changes, t-test p<0.001).

Table 2 presents changes in weight-related behaviours and psychological well-being from baseline to 12 months. The largest improvements were observed in pleasure shopping for clothes (baseline 4.51 ± 0.84 to 1.69 ± 0.89; mean change -2.82, 62.4% improvement), body image satisfaction (4.45 ± 0.82 to 1.82 ± 0.97; -2.63, 59.2%), and self-confidence (4.43 ± 0.89 to 1.88 ± 0.97; -2.55, 57.6%). Notable improvements occurred in health concerns (3.55 ± 0.96 to 1.84 ± 0.92; -1.71, 48.3%), social interactions (3.27 ± 1.30 to 1.39 ± 0.70; -1.88, 57.5%), and sexual life (3.71 ± 1.15 to 1.69 ± 0.98; -2.02, 54.4%). Food cravings showed modest improvement (1.27 ± 0.73 to 0.27 ± 0.45; -0.27, 17%). Smaller changes were observed for the ability to relax (0.55 ± 0.58 to 0.31 ± 0.62; -0.24, 44.4%) and concentrate (0.53 ± 0.92 to 0.29 ± 0.54; -0.24, 46.2%). Overall, 87.8-98.0% of participants demonstrated improvement or stability across assessed domains, indicating broad positive behavioural and psychological changes accompanying weight loss.

**Table 2:** Changes in weight-related behaviours and psychological wellbeing from baseline to 12 months Data are presented as mean (standard deviation) for continuous variables and n (%) for categorical variables. ᵃ All items used a 5-point Likert response scale (1 = never, 2 = rarely, 3 = sometimes, 4 = often, 5 = always), with higher scores indicating greater frequency or negative impact. Mean change was calculated as Month 12 mean minus Baseline mean, where negative values indicate improvement (reduction in negative behaviours/impacts). Percentage improvement was calculated as ((Baseline - Month 12) / Baseline) × 100. Questionnaire items were developed collaboratively with behavioural specialists and clinicians for internal programme evaluation and represent non-validated measures of behaviours and wellbeing

Domain	N	Baseline mean (SD)	Month 12 mean (SD)	Mean change	Percentage improvement of mean score(%)	Participants with improvement or no change N (%)	Participants with improvement N (%)
How often do you feel...^a^							
Food cravings	49	1.27 (0.73)	0.27 (0.45)	-1	79	48 (98.0%)	39 (79.6%)
Dissapointed while looking at photos of self	49	4.45 (0.82)	1.82 (0.97)	-2.63	59.2	48 (98.0%)	46 (93.9%)
Low because of your weight	49	3.57 (1.08)	1.53 (0.84)	-2.04	57.1	47 (95.9%)	45 (91.8%)
Concerned about your health	49	3.55 (0.96)	1.84 (0.92)	-1.71	48.3	48 (98.0%)	38 (77.6%)
How does your excess weight affect your...^a^							
Social interactions	49	3.27 (1.3)	1.39 (0.7)	-1.88	57.5	47 (95.9%)	41 (83.7%)
Self-confidence	49	4.43 (0.89)	1.88 (0.97)	-2.55	57.6	48 (98.0%)	46 (93.9%)
Pleasure shopping for clothes	49	4.51 (0.84)	1.69 (0.89)	-2.82	62.4	48 (98.0%)	48 (98%)
Energy levels	49	3.57 (1.02)	1.59 (0.86)	-1.98	55.4	47 (95.9%)	43 (87.8%)
Sense of self-control	49	1.27 (0.64)	0.29 (0.46)	-0.98	77.4	48 (98.0%)	37 (75.5%)
Sexual life	49	3.71 (1.15)	1.69 (0.98)	-2.02	54.4	46 (93.9%)	42 (85.7%)
Exercise levels	49	3.04 (1.14)	1.43 (0.79)	-1.61	53	47 (95.9%)	37 (75.5%)
Ability to relax	49	0.55 (0.58)	0.31 (0.62)	-0.24	44.4	47 (95.9%)	14 (28.6%)
Physical comfort	49	2.71 (1.15)	1.35 (0.6)	-1.37	50.4	47 (95.9%)	35 (71.4%)
Ability to concentrate	49	0.53 (0.92)	0.29 (0.54)	-0.24	46.2	43 (87.8%)	12 (24.5%)

Blood pressure outcomes

**Figure 3:**
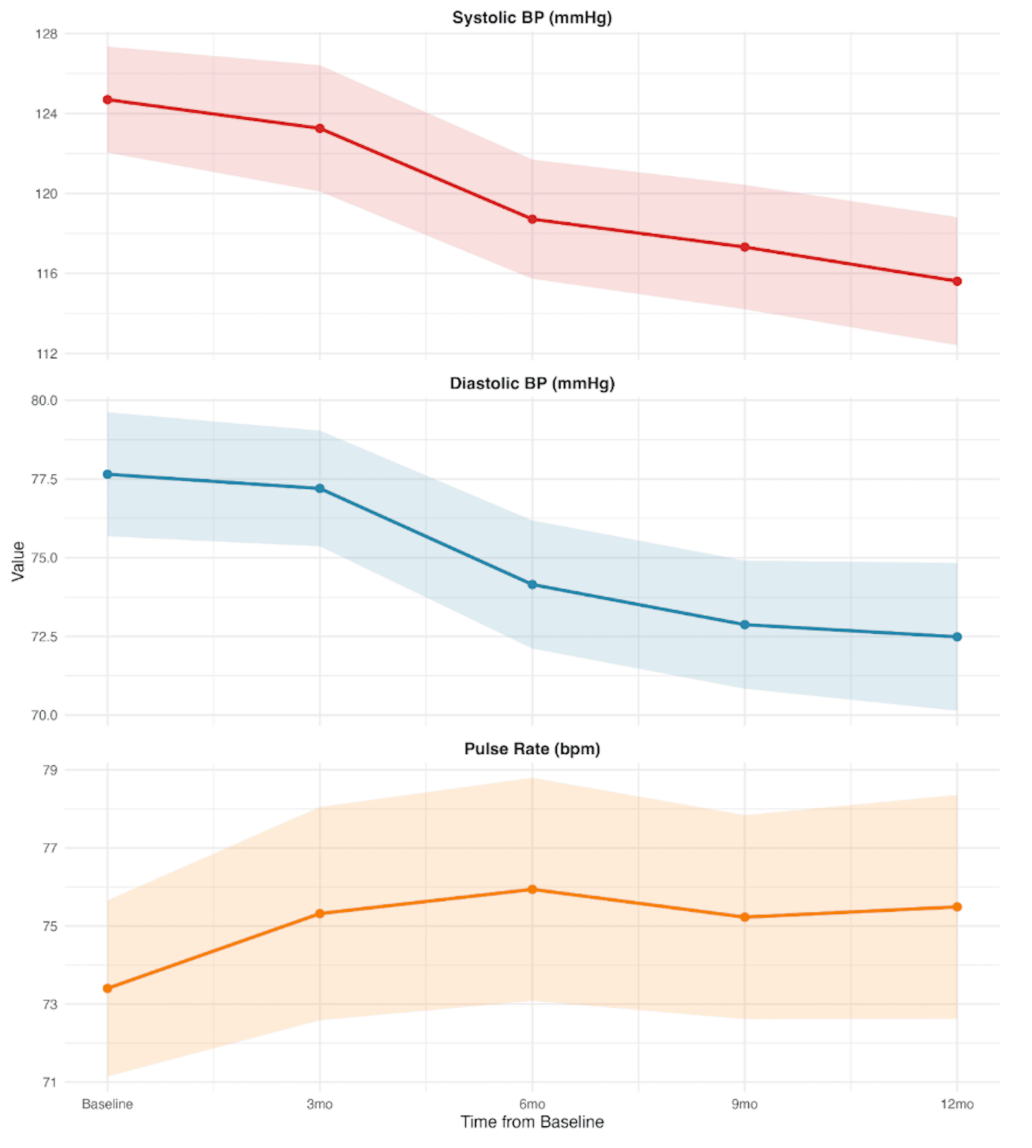
Change in blood pressure and mean pulse rate Trajectories of systolic and diastolic blood pressure (BP) and pulse rate (beats per minute (bpm)), mean values with 95% confidence intervals over 12 months are shown. Both systolic (red line) and diastolic (blue line) BP show progressive reductions at 12 months, while pulse rate (orange line) remains relatively stable with minor fluctuations.

Both blood pressure parameters improved significantly at 12 months (Figure 3). From baseline, systolic blood pressure decreased by 9.07 mmHg (95% CI 5.52 to 12.61, p<0.001) and diastolic blood pressure decreased by 4.90 mmHg (95% CI 2.53 to 7.26, p<0.001). Mean pulse rate increased slightly by 2.62bpm by 12 months, however this change was not deemed clinically significant (95% CI 0.16 to 5.08, p<0.05).

Side effects

**Figure 4:**
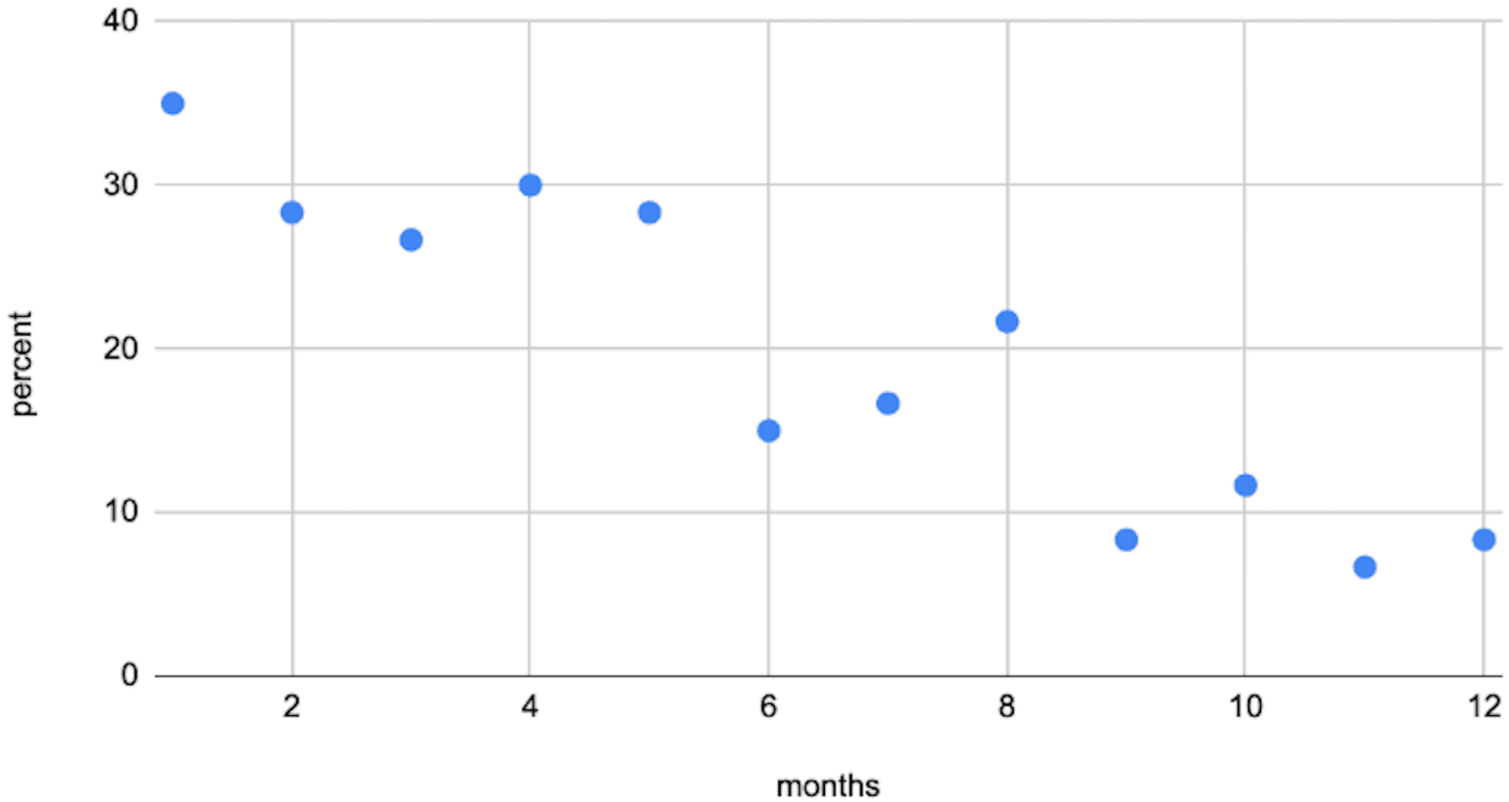
Prevalence of nausea. Prevalence of nausea over 12 months among the cohort. The proportion of individuals reporting nausea decreased from 35% within the first month to 8% by month 12.

Patients reported side effects through routine questionnaires, reflecting their treatment tolerability over time. The most commonly reported side effect was nausea, followed by constipation and headache. Other frequently reported symptoms were diarrhoea and fatigue, indicating that most symptoms were gastrointestinal by nature. The proportion of individuals reporting side effects decreased with time and across all symptoms. For instance, nausea frequency, the most common side effect, decreased from 35% within the first month to 8% at month 12. Constipation and headaches also decreased, from 33% to 10% and from 28% to 5% respectively (Figure 4).

Laboratory blood results

Longitudinal mixed-effects models revealed significant time-driven improvements across several cardiometabolic blood markers. The addition of demographic covariates (age, gender, BMI) did not significantly improve model fit (p>0.05) or alter the temporal trajectory, indicating improvements were independent of these baseline characteristics. No statistically significant changes were observed in thyroid function (free T4 and TSH) or iron stores (ferritin).

HbA1c

**Figure 5:**
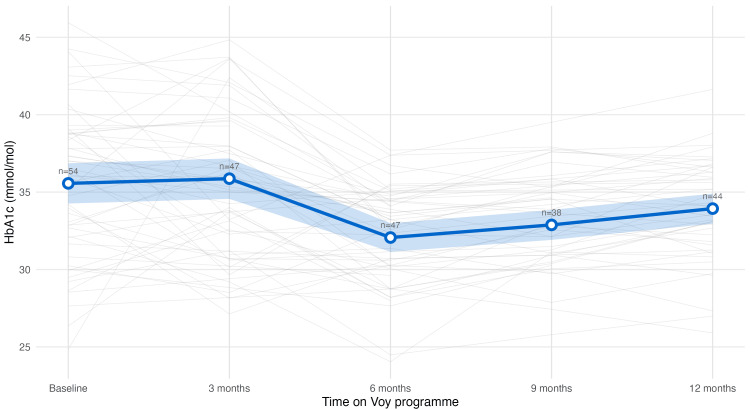
HbA1c trajectory Trajectory of glycated haemoglobin (HbA1c) levels over 12 months among the cohort. Grey lines: individual participants' values, blue line: population mean, blue shade: 95% confidence intervals. Mean HbA1c decreased from baseline to month six, followed by a modest rise through month 12.

At baseline, mean HbA1c was 35.6 ± 4.9 mmol/mol, within normoglycemic. 9.3% (n=5) participants had prediabetic HbA1c levels (≥ 42 mmol/mol). After 12 months, HbA1c levels reduced by 2.4 mmol/mol (95% CI 1.41 to 3.53, p<0.001) (Figure 5), and all participants with prediabetic HbA1c levels normalised to a healthy range (<42 mmol/mol).

Lipid Profile

Baseline lipid levels were characterised by slightly elevated, above desired levels, with values for total cholesterol equal to 5.73 ± 1.40 mmol/L, mean LDL cholesterol to 3.57 ± 1.14 mmol/L and total cholesterol/HDL ratio to 4.36 ± 1.71.

**Figure 6:**
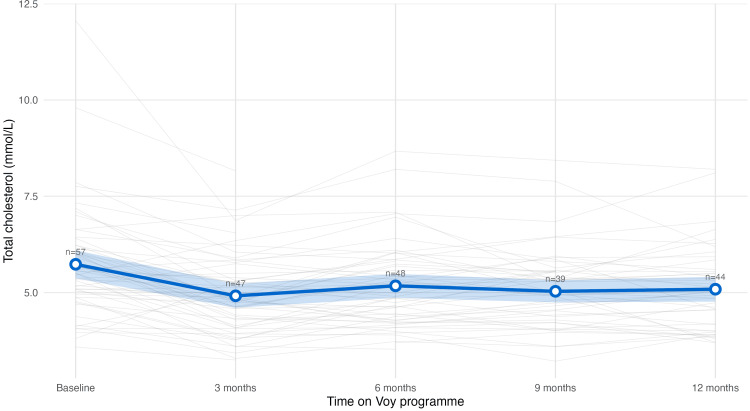
Total cholesterol trajectory Trajectory of total cholesterol levels over 12 months among the cohort. Grey lines: individual participants’ values, blue line: population mean, blue shade: 95% confidence intervals. Mean total cholesterol levels decreased from baseline to month three and remained relatively stable through month 12.

At 12 months, total cholesterol levels were significantly reduced, with a decrease of 0.46 mmol/L (95% CI 0.20, 0.73, p<0.01) (Figure 6). Of the at-risk participants, 29% achieved full normalisation, and 69% showed directional improvement by 12 months.

**Figure 7:**
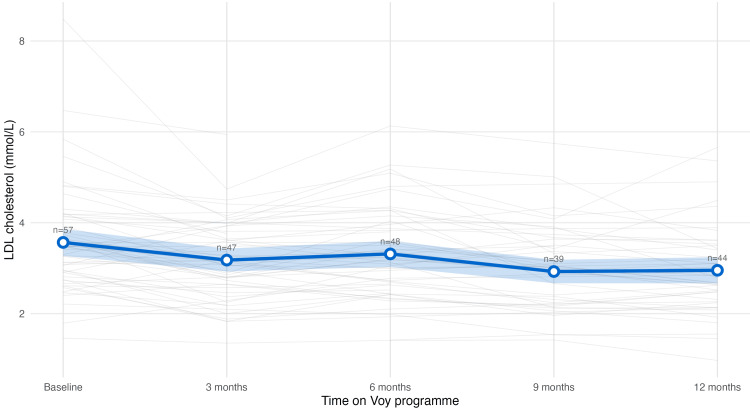
LDL cholesterol trajectory Trajectory of low-density lipoprotein (LDL) cholesterol levels over 12 months among the cohort. Grey lines: individual participants' values, blue line: population mean, blue shade: 95% confidence intervals. LDL cholesterol levels showed a steady reduction from baseline through month 12.

For LDL cholesterol, a significant decrease of 0.52 mmol/L (95% CI 0.31 to 0.72, p<0.001) was observed (Figure 7). Of the participants at-risk at baseline, 31% crossed into the healthy range, and 72% showed directional improvement. 

**Figure 8:**
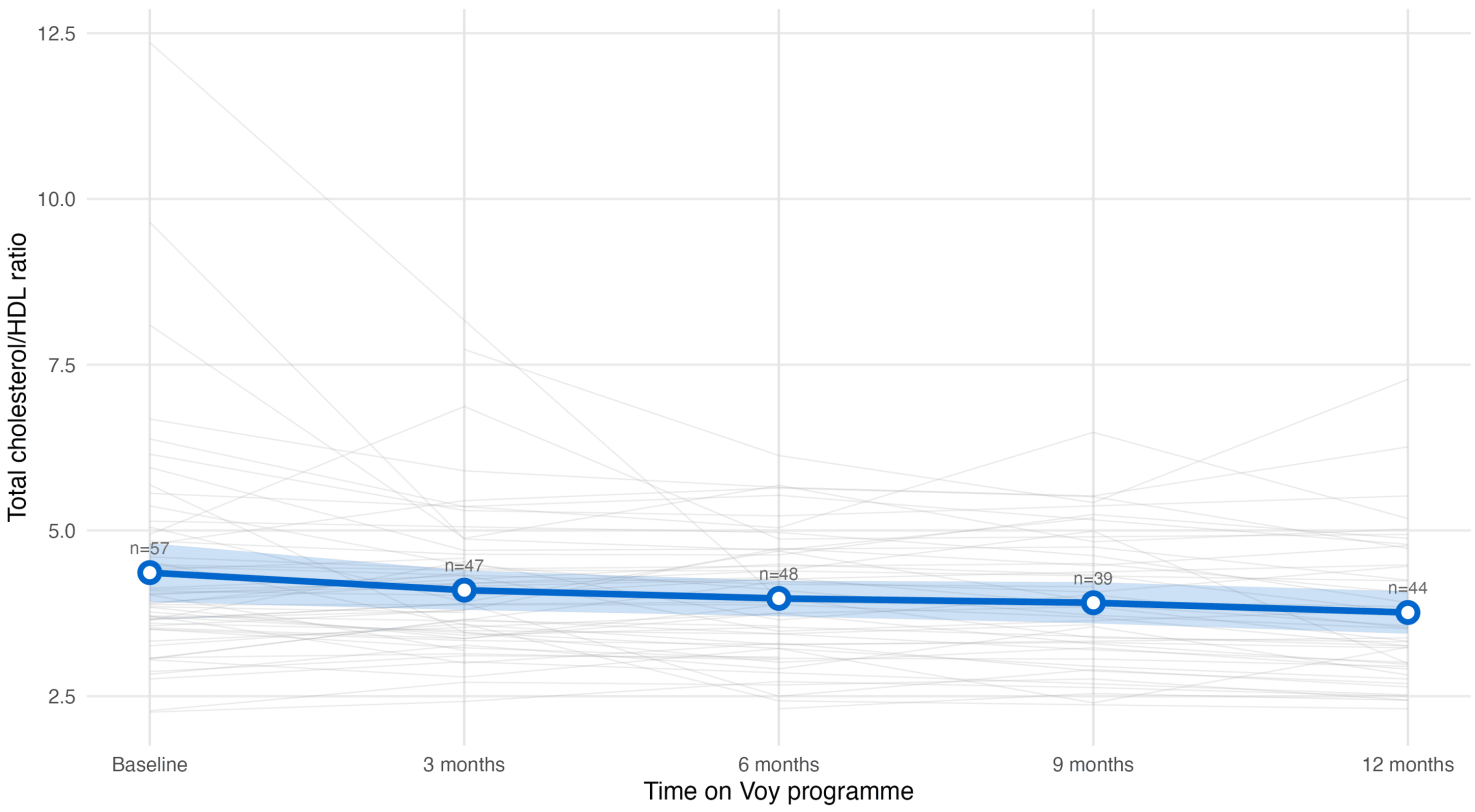
Total cholesterol/HDL ratio trajectory Trajectory of total cholesterol/high-density lipoprotein (HDL) ratio levels over 12 months among the cohort. Grey lines: individual participants' values, blue line: population mean, blue shade: 95% confidence intervals. The cholesterol/HDL ratio declined gradually across the study period of 12 months.

Total cholesterol/HDL ratio significantly decreased by 0.62 units (95% CI 0.22 to 1.03, p<0.01) (Figure 8). One-third (33%) of participants who were at-risk at baseline achieved healthy ratios. No statistically significant change in HDL cholesterol was observed over the 12-month period (p=0.712).

Thyroid Function (Free T4 and TSH)

Thyroid function remained stable, with no significant changes for both free T4 and TSH levels (p>0.05).

Iron Stores (Ferritin)

There was a slight upward trend in ferritin levels, reaching +15.0 µg/L at 12 months, but it did not reach statistical significance. The analysis showed an age effect, with ferritin levels increasing with age - 4.2 µg/L higher (p<0.0001) for every additional year of age.

## Discussion

This service evaluation demonstrated that the Voyager programme attained clinically meaningful weight loss outcomes, alongside favourable changes in a wide range of anthropometric and biochemical outcomes over 12 months. The mean 12-month weight reduction of 22.7% (95% CI 20.92% to 24.48%, p<0.001) is consistent with outcomes reported in randomised clinical trials such as SURMOUNT, and compares favourably against other real-life weight management programmes with tirzepatide [18, 19]. The study’s findings regarding trends in biochemical markers such as lipid profiles on GLP-1 therapy align with existing evidence [18]. Most importantly, the programme was associated with broader, holistic advancements in physical and mental health, including reductions in binge eating and depressive symptoms. Therefore, we focus our discussion on these aspects.

Binge eating tendencies and mental well-being are intrinsically interconnected with physical health outcomes for people living with obesity [20]. In a systematic review by Pierret et al., they demonstrated that addressing psychological factors such as depression, emotional eating and body image can improve both weight management outcomes and overall quality of life [21]. This relationship between binge eating tendencies, mental health issues and weight is bidirectional: excess weight can impair wellbeing and exacerbate binge eating behaviours, while adverse mental states reinforce maladaptive eating patterns that contribute to weight gain and obesity [20, 22]. Luppino and colleagues showed in their meta-analysis that people living with obesity are 55% more likely to develop depression, and those living with depression are 58% more likely to develop obesity (OR, 1.58; 95% CI, 1.33-1.87; p<0.001) [22].

Holistic care has also been underscored by regulatory and clinical guidance, including National Institute for Health and Care Excellence (NICE), which stresses that effective management of obesity as a chronic, multifactorial condition must address both physical and psychological needs [23]. Additionally, omitting the psychological aspects and focusing solely on weight loss provides only a partial assessment of the effectiveness of digital weight loss programmes. Evaluations focusing on weight provide an incomplete understanding of whether participants developed healthier relationships with food or improved their psychological functioning. Although evidence is limited, prior studies on tirzepatide and other weight loss medications suggest adjunct psychological and behavioural gains [21, 24], both in the management of binge eating [8, 9, 18, 25] and depressive symptoms [26]. These findings suggest that anti-obesity medication may exert favourable effects on metabolic and psychological outcomes, although further research is needed to confirm these associations [21].

Binge eating tendencies are common among individuals with obesity [25]. The Binge Eating Score (BES) is a validated self-report instrument measuring emotional and behavioural symptoms linked to emotional and behavioural tendencies around binge eating [27]. In this study, participants demonstrated a reduction in binge eating score by 13 points at three months (95% CI 9.19 to 17.05, p<0.001) of treatment and 18 points at 12 months (95% CI 14.5 to 21.67, p<0.001), suggesting a positive long-term impact of the intervention on emotional eating. These reductions are greater than those reported in previous studies that used GLP-1 analogues, including liraglutide, dulaglutide and semaglutide [8-10]. The open-label studies of liraglutide and dulaglutide reported mean or median reduction in BES scores that ranged from nine (p<0.001) to 12 (p<0.0001) points at 12 weeks of treatment [8, 10]. Similarly, a retrospective cohort study of semaglutide reported a mean reduction of 14 points, calculated over a variable follow-up duration [9]. Therefore, our findings align with the literature [28], presenting tirzepatide and other anti-obesity medications as potential agents in supporting binge eating symptoms during treatment for obesity, likely through action on the food reward circuit and regulation of the appetite rather than through cognitive restriction [28]. The magnitude of improvement observed in BES scores in this evaluation (13 points at three months, 18 points at 12-months) may suggest that the digital components of the weight loss programme, together with the tirzepatide treatment, may reinforce positive changes in emotional eating behaviours over time, in parallel with the observed weight loss trajectory.

Putative mechanisms underlying this effect could be attributed to physiological, behavioural and psychological processes influenced by distinct components of the intervention. Firstly, the tirzepatide pharmacologically acts on gut and brain pathways, improving appetite and satiety regulation and reducing hunger [6]. By impacting the reward circuits, it may also attenuate self-control and food cravings or “food noise”, which may further lower the susceptibility to binge eating symptoms [6]. Secondly, the behavioural elements of the digital programme, including health coaching and clinical support, and access to the digital app with educational materials and progress tracking, may have positively impacted self-awareness, accountability and sustained motivation, supporting sustained changes in binge eating patterns [29]. Indeed, evidence indicates that active support from a multidisciplinary team of clinicians and dietitians, combined with educational support, has been shown to improve mental health, quality of life, and weight outcomes in obesity management programmes [20]. For the optimum treatment of obesity, leading to holistic results and broad improvements in health and wellbeing, a comprehensive care approach is essential, one that integrates behavioural and dietary support with clinical management, digital health tools, and other complementary interventions [11].

Limitations and future directions

Despite examining a range of relevant outcomes, several limitations of the study should be acknowledged. As a single-arm study without a control group, the study cannot establish causality and may be subject to confounding. Furthermore, the modest sample size (N=60) limits statistical power to detect subgroup effects, and the self-selected nature of participants who typically enrol in digital weight loss programmes like Voy (predominantly female and White British/Irish) may restrict generalisability to broader or more diverse populations beyond those enrolled in this specific programme. With 90% of the sample being women, a common imbalance in gender proportions seen in other weight loss trials and studies, where the female participants are more likely to partake in DWLS; however, the gender disparity contributes to the selection bias, further limiting the generalisability of the results to the wider UK population [12]. Future research should aim for more balanced recruitment and consider analytical approaches, such as propensity score methods or weighting, to mitigate imbalance and reduce the risk of biased outcome estimates. Participants were also likely to be more motivated, health-literate, or financially able to pay for such services than average, which may further contribute to selection bias. Moreover, outcomes such as mental health, binge eating, weight, and anthropometric measures were self-reported, and thus subject to recall bias, social desirability bias, and human error. This could manifest as underreporting of binge episodes and weight gain, and over-reporting of weight loss, further skewing the results. Finally, attrition bias might have affected the results. This is because the final dataset's representativeness could be compromised by differential dropout, potentially related to participants' success, or lack thereof, on the weight loss programme.

Further research should prioritise the collection of longitudinal data beyond the 12-month period. This would facilitate a better understanding of the long-term effects of the programme on the mental health and wellbeing as well as enable insight into the potential maintenance period for the patients seeking to sustain, rather than continue to lose, weight.

## Conclusions

This study provides useful real-world results regarding the ability of digital weight loss services to attain marked improvements in weight, biochemical risk markers, binge eating behaviours, and mental health. The primary outcome, percentage total body weight loss at 12 months, showed improvements comparable to landmark weight loss trials. Other outcomes, including improvements in anthropometric measurements, laboratory results, blood pressure, side effects, and most importantly, binge eating and depressive symptoms, showcase the significant potential of digital weight loss services. Overall, this service evaluation offers a unique insight into the broader impacts of a weight loss programme beyond profound weight change. Further large-scale research focused on prospective studies and randomised control trials is needed to determine optimal dosing, identify which anti-obesity medications are most effective for treating binge eating and depressive symptoms, and establish which elements of the digital weight loss programme are most crucial in improving the behavioural and psychological condition of the patients. Given the limited male representation in this sample, future research should prioritise recruiting male participants to better examine gender disparities and potential differences in responses to the medication and programmes’ digital components. It should also collect longer-term data to clarify the programme’s lasting effects on mental health, wellbeing and weight maintenance.
